# The London

**Published:** 1907-09-07

**Authors:** 


### THE LONDON.

The student who desires to take the degree of M.B. of the London University has to pass the University Matriculation Examination, held thrice yearly in January, September, and June. The Matriculation is accepted as a preliminary examination for graduation or qualification in medicine, provided the candidate has taken Latin as one of the optional subjects, and formerly no other examination exempted from the matriculation so far as degrees of the University were concerned. Recently, however, the Senate has seen fit to rule that several other examinations may be accepted instead of the usual Matriculation under certain conditions. For such exempted candidates standing, in University language, will date from the time of registration, and, except under very special circumstances when extraordinary permission may be granted by the Senate on application, no matriculate may proceed to a degree within three years of the date of his matriculation.

### The Preliminary Studies.

Assuming that the student, then, has been registered as a matriculated student of the University, either upon passing the examination or after having been exempted from it, he is able to devote himself to medical study. He must attach himself to one or other of the various medical schools, and devote himself during his first year to mastering the subjects—chemistry, biology, and physics—required for his Preliminary scientific or first professional examination. It will take him a full year to work up these subjects, though he may pass the examination in parts if he prefers. Many candidates, instead of doing the Preliminary scientific, work somewhat more widely and attempt to take the Intermediate scientific, which, provided the candidate has passed in the requisite subjects, exempts from the Preliminary scientific, and at the same time enables the student to proceed to his B.Sc. degree if he chooses.

### The Intermediate Examination.

The student is admitted to the Intermediate examination, the subjects of which consist of anatomy, physiology, and pharmacology, two years after having passed Part I. of the Preliminary. Time spent in working at the subjects of anatomy and physiology is not recognised by the University until the student has passed the Preliminary. His second and third years will thus be spent in work in the dissecting room and physiological laboratory. Here he will be able to work more broadly and more minutely at the subjects than the Conjoint student is able to do. Thus he may be able to dissect the whole body, and to do several parts twice over; he can also devote his attention to dissecting the brain and spinal cord—subjects which Conjoint students are usually unable to give much time to. In physiology he can do more work in histology and experimental physiology, while his lectures in pharmacology will prove of great service to him afterwards. The examination itself is of average difficulty. The candidate is required to dissect a part, and is examined *viva voce* in each subject, with written papers in each subject and practical work in physiology. If the student has been diligent at his work he should not have the slightest difficulty in passing his intermediate examination well after the two years' period.

### The Final Years.

Having passed his "Inter.," the student enters the wards, generally in the capacity of clerk, and goes through the ordinary routine duties of dresser and extern obstetric assistant. Two years after passing the Inter. he is allowed to enter for his final examination, which consists of the usual professional subjects. The final is by no means a stiff examination; compared with the Preliminary examination, it is even an easy one. Special attention must be paid to a few subjects. Thus the student will do well to keep himself acquainted with pathology and morbid anatomy; midwifery and forensic medicine, also, are subjects in which many candidates fail. But the two years' ward work, supplemented by reading and attendance at the outpatient department, will enable the student to acquit himself well at the final. The last examination is written, oral, and practical. The candidate is required to examine and report on selected cases, to do practical bacteriological work, and to be acquainted with the essentials of operative surgery, though actual operations on the dead subject are not demanded.

